# The comparative effectiveness and efficiency of cognitive behaviour therapy and generic counselling in the treatment of depression: evidence from the 2^nd^ UK National Audit of psychological therapies

**DOI:** 10.1186/s12888-017-1370-7

**Published:** 2017-06-09

**Authors:** Jo Pybis, David Saxon, Andy Hill, Michael Barkham

**Affiliations:** 1 Research Office, British Association for Counselling and Psychotherapy, BACP House, 15 St John’s Business Park, Lutterworth, Leicestershire, LE174HB UK; 2 Centre for Psychological Services Research, ScHARR, University of Sheffield, Sheffield, UK; 3 Centre for Psychological Services Research, Department of Psychology, University of Sheffield, Sheffield, UK

**Keywords:** CBT, Counselling, Psychological therapy, Depression, Effectiveness, Efficiency

## Abstract

**Background:**

Cognitive Behaviour Therapy (CBT) is the front-line psychological intervention for step 3 within UK psychological therapy services. Counselling is recommended only when other interventions have failed and its effectiveness has been questioned.

**Method:**

A secondary data analysis was conducted of data collected from 33,243 patients across 103 Improving Access to Psychological Therapies (IAPT) services as part of the second round of the National Audit of Psychological Therapies (NAPT). Initial analysis considered levels of pre-post therapy effect sizes (ESs) and reliable improvement (RI) and reliable and clinically significant improvement (RCSI). Multilevel modelling was used to model predictors of outcome, namely patient pre-post change on PHQ-9 scores at last therapy session.

**Results:**

Counselling received more referrals from patients experiencing moderate to severe depression than CBT. For patients scoring above the clinical cut-off on the PHQ-9 at intake, the pre-post ES (95% CI) for CBT was 1.59 (1.58, 1.62) with 46.6% making RCSI criteria and for counselling the pre-post ES was 1.55 (1.52, 1.59) with 44.3% of patients meeting RCSI criteria. Multilevel modelling revealed a significant site effect of 1.8%, while therapy type was not a predictor of outcome. A significant interaction was found between the number of sessions attended and therapy type, with patients attending fewer sessions on average for counselling [M = 7.5 (5.54) sessions and a median (IQR) of 6 (3–10)] than CBT [M = 8.9 (6.34) sessions and a median (IQR) of 7 (4–12)]. Only where patients had 18 or 20 sessions was CBT significantly more effective than counselling, with recovery rates (95% CIs) of 62.2% (57.1, 66.9) and 62.4% (56.5, 68.0) respectively, compared with 44.4% (32.7, 56.6) and 42.6% (30.0, 55.9) for counselling. Counselling was significantly more effective at two sessions with a recovery rate of 34.9% (31.9, 37.9) compared with 22.2% (20.5, 24.0) for CBT.

**Conclusions:**

Outcomes for counselling and CBT in the treatment of depression were comparable. Research efforts should focus on factors other than therapy type that may influence outcomes, namely the inherent variability between services, and adopt multilevel modelling as the given analytic approach in order to capture the naturally nested nature of the implementation and delivery of psychological therapies. It is of concern that half of all patients, regardless of type of intervention, did not show reliable improvement.

**Electronic supplementary material:**

The online version of this article (doi:10.1186/s12888-017-1370-7) contains supplementary material, which is available to authorized users.

## Background

In England, the publication of the Depression Report [1] set in motion a sea change in the policy and implementation of psychological services in the country. The report argued that there were effective psychological interventions available, essentially Cognitive Behaviour Therapy (CBT), but that services were not implementing evidence-based therapies according to National Institute for Health and Care Excellence (NICE) guidance. In order to respond to the high rates of depression and anxiety and combined with an economic argument that the savings on benefits would balance the costs of the programme, the UK government invested an initial £33 million into the Improving Access to Psychological Therapies (IAPT) programme. It was then rolled out in 2008 with the aim of providing evidence-based psychological therapies to adults in England experiencing anxiety disorders and depression [1]. Further investments of £110 million and £173 million were provided in 2009/10 and 2010/11 [2].

In line with the NICE Guidelines for Depression in adults [3], IAPT adopted a stepped care model where patients are assessed (step 2) by Psychological Wellbeing Practitioners (PWPs) with the expectation that they will treat the majority of patients. Only those patients who are severe on referral or who show no improvement within a period of time from a step two intervention are ‘stepped up’ to a high-intensity intervention (step 3) [4]. Within the same guidelines, NICE recommended CBT as the frontline psychological therapy. By contrast, counselling was viewed as a second line intervention where counsellors were required to ‘Discuss with the person [patient] the uncertainty of the effectiveness of counselling….in the treatment of depression’ (p.16).

Despite this statement, various studies have examined the effectiveness of counselling, often referred to as non-directive supportive therapy (NDST), as a treatment for adult depression [4, 5]. In all of these studies non-directive supportive therapy is an unstructured therapy where the therapist refrains from giving advice or making interpretations and the therapy typically is not aimed at providing solutions or acquiring new skills [6, 7].

In terms of evidence from meta-analytic studies, one meta-analysis marshalled data from 34 randomized controlled trials of brief psychological therapies of adult patients with anxiety, depression or mixed common mental health problems treated in primary care compared to primary care treatment as usual [8]. The authors reported brief CBT, counselling, and problem solving therapy all to be effective treatments in primary care, but effect sizes were lower compared to longer length treatments [8]. In another meta-analysis, 67 studies were analysed [9]. Results suggested that treatment with non-directive supportive counselling was less efficacious than pharmacotherapy. In an earlier meta-analysis of 31 studies, Cuijpers and colleagues found non-directive counselling to be effective in the treatment of depression in adults but that it was somewhat less effective than other more structured psychological treatments [7]. However, all the effect size differences were small, and in one study were no longer present after controlling for researcher allegiance [6]. Additionally, as highlighted by Cuijpers and colleagues, the types of therapies grouped together as ‘non-directive supportive therapy’ range from client centred therapy to support groups and there may be great variability in what is being provided [7].

In terms of evidence from randomised controlled trials (RCTs), a comparison between non-directive counselling and cognitive behaviour therapy for mixed anxiety and depression and also for depression alone reported no significant difference in outcomes for the two therapies [10]. A subsequent reanalysis of the subsample meeting a diagnosis of depression only found similar results with both therapies being superior to usual General Practice care at 4 months but not at 12 months [11]. More recently, a trial exploring the effectiveness of acupuncture and counselling in primary care reported statistically significant benefits at 3 months associated with both interventions when provided alongside usual care [12].

The literature would therefore suggest that counselling is effective and that where differences with CBT occur they are small (i.e., an effect size <.2). Accordingly, there appears little supporting evidence for continuing to state that generic counselling should be regarded as a second-line treatment for the treatment of depression. However, concerns about the lack of a single standardized form of counselling led to initiatives to devise a form of counselling that was based on a combination of generic and specific competences within humanistic therapies as well as drawing on evidence from randomised controlled trials. This concern led to the development of a blended form of person-centred experiential therapy termed Counselling for Depression (CfD) that combined person-centred counselling with key aspects of emotion focused therapy drawing on experiential models [13].

Training in CfD has been delivered since 2011 and the number of funded CfD training places has increased annually [14]. A recent IAPT report showed CBT and Counselling for Depression (CfD) to be the most widely accessed interventions with 57% of patients accessing CBT and 23% accessing CfD [15]. The first published outcomes following the rollout of CfD training presented ‘recovery rates’ by therapy type and problem descriptor [15]. Outcome recovery rates were comparable for CBT (45.9% for depression, 49% for anxiety) and CfD (47.6% for depression, 46.7% for anxiety). Interestingly, the average number of sessions attended for CBT (*N* = 7.1) was greater than for CfD (*N* = 5.9).

However, much of the IAPT counselling provision is still delivered by qualified counsellors who are not yet trained in CfD. As reported in the 2015 Adult IAPT Workforce Census Report, whilst CBT provided 61.9% of the workforce, CfD accounted for only 5.7% whereas generic counselling still comprised 11.1% of the IAPT workforce [16]. Moreover, data collected as part of the second independent National Audit of Psychological Therapies [17] indicated generic counselling to be the second most widely available psychological therapy in IAPT, with 29.2% of patients receiving counselling compared to 69.3% receiving CBT. The other approved interventions accounted for less than 2% of the total provision [18]. In light of the continued practice of generic counselling, it would be important to determine its comparative effectiveness as practised immediately prior to the roll out of CfD within the IAPT service particularly as, to date, there is no evidence-base derived from trials attesting to the efficacy of CfD itself or whether it has yielded improved outcomes or a more robust evidence-base.

Pre-dating the national rollout of CfD training, evaluations of the initial years of the IAPT programme provided preliminary data on the comparative outcomes of the therapies provided by IAPT [19, 20]. An initial evaluation of the first wave of the IAPT programme (2008/2009) aimed to determine how successfully the commitments to accessibility, provision of NICE-recommended psychological therapies, and outcome monitoring were progressing. Outcomes were comparable for both CBT and generic counselling with approximately 40% of people moving to recovery (i.e., below caseness at discharge) in each intervention [19]. A secondary analysis using a dataset taken from 32 of the initial roll out IAPT sites used logistic multiple regression to investigate the variability in performance of these two therapies and how the variability between sites and patients affected patients’ recovery. Recovery rates were found to be comparable to those reported in the initial evaluation, with approximately 40% of patients moving to recovery for both interventions [20]. However, only a relatively small number of sites (*N* = 18 and 32 respectively) were included in these analyses of IAPT data and neither accounted for differences between patients or differences between sites.

The use of rates of reliable and clinically significant improvement and reliable improvement only are the current primary methods for reporting outcomes in IAPT [21, 22]. Each of these two procedures is appropriate for specific situations: reliable and clinically significant improvement focuses on only patients who meet clinical threshold at intake, while reliable improvement does not require this criterion and can therefore be applied to virtually all patients referred to a clinical service. However, irrespective of which criterion is used, these procedures do not acknowledge the nested nature of data, thereby leading to simplistic ranking of services in which patient casemix is not taken into account.

Accordingly, we sought to build on the reported findings by accessing IAPT data collected as part of the second round of the National Audit of Psychological Therapies [17] in order to test one primary proposition, namely that the variability between service providers would have a significant effect on patient outcomes while the therapy they received, counselling or CBT, would not. Such a finding would support the argument that the focus of research attention has been misplaced by investing in the apparent superiority of one model of psychological intervention over another rather than investing in the study of the variability in patient outcomes between different psychological therapy sites or providers. A secondary aim was to consider any differences in the effectiveness of the two therapies in relation to the number of sessions that patients attend.

## Methods

### Study sample

Access to data from IAPT services participating in the second National Audit of Psychological Therapies (NAPT) was granted from the Healthcare Quality Improvement Partnership (HQIP). The aim of the audit was to evaluate the quality of treatment and care received by people seeking psychological therapy services for common mental health disorders in England and Wales as a basis for service improvement. Reflecting the focus on services and service outcomes, data was not collected on therapists and therapists were not recognised or identifiable in the data. The baseline audit was carried out in 2010 and published in November 2011 [23] with a second round of the audit conducted 18 to 24 months after the baseline to determine whether performance had improved [17]. The current study used only IAPT data from the second audit of all NHS-funded psychological therapy services for adults in primary and secondary care in England and Wales. Data were requested on all patients discharged from services between 1st July and 31st October 2012.

**Fig. 1:**
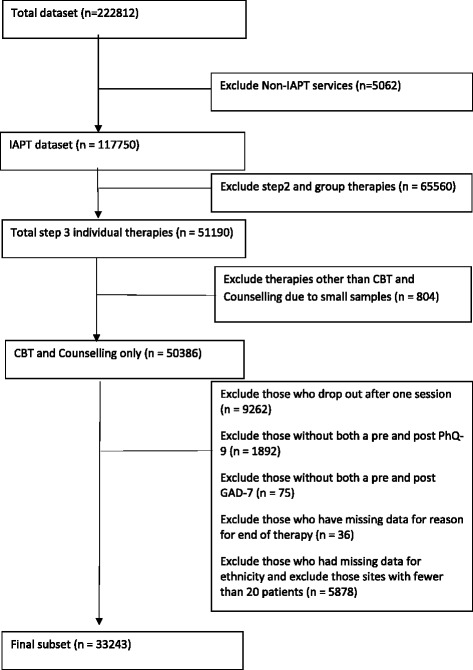
Flowchart

The full NAPT retrospective case record comprised data from 122,812 individual patients from both IAPT and non-IAPT sites. IAPT services (*N* = 121) provided 117,750 case records. In order to determine a change score as well as excluding patients who only received an initial assessment (i.e., one session), patients were excluded if they did not receive two or more sessions of a step 3 therapy for depression (CBT or counselling), or had missing data for important variables; pre or post-therapy outcome measures, ethnicity (used as a proxy measure of socio-economic deprivation) and the reason for treatment ending. Further, because we were interested in both the variability between services, the ‘site effect’, and the reliability of model coefficients, services that provided data on fewest patients were excluded while maintaining the recommended number of services to reliably estimate site effects [24, 25]. These exclusions resulted in a study sample of 33,243 patients (CBT: *n* = 23,595; counselling: *n* = 9648) treated at 103 sites (Fig. 1). The number of patients per site ranged from 21 to 1858. Patients were allocated to treatment through standard routine practice procedures. Such decision rules vary across services but will include availability of a practitioner regardless of their theoretical orientation, assignment by a step 2 practitioner in terms of the issues identified by the patient (e.g., relationship issues being assigned to counselling and specific problems being assigned to CBT), or patient stated preferences. However, there are no national guidelines as to which therapy a patient should be referred to other than the previously stated CBT as the frontline treatment and counselling as a second line treatment.

### Sample characteristics

The patient demographic profile of the study dataset was comparable to that of all patients accessing any step 3 psychological intervention within the full NAPT dataset [17] with 66% female, 83.7% white British and a mean age of 40.99 (SD 13.86) years. A primary presenting problem was recorded for 21,105 (63.5%) patients, with the largest categories being depressive episode (15.8%), anxiety and depression (15.7%), and Generalised Anxiety Disorder (7.1%). Other problems included recurrent depressive episode (5%), panic disorder (3.1%), post traumatic stress disorder (PTSD) (3.1%), and Obsessive Compulsive Disorder (OCD) (3%). The proportion of patients that completed therapy was similar in both treatments: 68.2% for CBT and 69.4% for counselling (*χ* ^2^ (1) = 4.512 *p* = 0.034).

### Therapists

Therapist level data were not available in the NAPT dataset. However, all practitioners delivering CBT or generic counselling within IAPT are typically trained to post-graduate diploma level and receive regular supervision as set out within IAPT guidance.

### Psychological interventions

In terms of defining these two forms of psychological therapy, CBT is based on the premise that the way we feel is affected by our thoughts, beliefs and by how we behave. People become depressed for many different reasons, for example due to stress or relationship breakdown. Depression tends to trigger negative thoughts, which can increase depression and lead to negative behaviour. Changing how a person thinks when depressed, and what they do as a result, can also change how they feel. CBT involves planning practical exercises or experiments, encouraging people to engage in activities and to write down their thoughts and problems for discussion during therapy. CBT can also involve problem-solving and learning how to deal with worry or with difficult memories [26].

With regards to generic counselling, it is more difficult to define as there are many different theoretical approaches that a counsellor could be trained in, meaning they may work with patients in different ways. Although the National Audit of Psychological Therapies did not require practitioners to provide any details about the form of counselling they delivered, ‘counselling’ was an option alongside specific modalities including CBT, Interpersonal Therapy, Solution Focused Therapy, and Cognitive Analytic Therapy. Therefore it can be assumed that practitioners who selected ‘counselling’ were providing something different to those specific modalities. Many counsellors practise in an integrative manner where they bring skills and knowledge from training underpinning different forms of therapy. Typically, counselling involves a series of formal sessions, usually six to twelve sessions, where the therapist and the client talk about the client’s issues and feelings. Therapy may involve talking about life events, feelings, emotions, relationships, and ways of thinking and patterns of behaviour. The therapist will listen, encourage and empathise, but will also challenge in order to help the client see their issues more clearly or in a different way. Counselling is not about giving advice or opinions, nor is it a friendly chat with a friend. The therapist helps the client to understand themselves better and find their own solutions to resolve or cope with their situation [27].

### Outcome measures

Because of the high level of comorbidity between depression and anxiety, the IAPT programme uses two primary outcome measures: the Patient Health Questionnaire-9 (PHQ-9) [28] and the Generalised Anxiety Disorder-7 (GAD-7) [29].

The Patient Health Questionnaire-9 (PHQ-9) [28] is a brief 9-item self-report measure of depression derived from the Patient Health Questionnaire and constitutes a self-administered version of the PRIME-MD. It is a measure designed to assist medical practitioners making criteria-informed diagnoses of DSM-IV disorders commonly experienced by medical patients. The measure uses a 4-point Likert-type scale with scores ranging from 0 (*“not at all”*) to 3 (*“Nearly every day”*) with total PHQ-9 scores ranging from 0 to 27. The time frame captured by the PHQ-9 comprises a two-week time period prior to completing the questionnaire. Scores of 10 and above on the PHQ-9 are demarcated as clinical scores and these scores showed criterion validity when assessed against mental health professional interviews [28]. Severity bands (and range of scores) are as follows: minimal/mild (0–9), moderate (10–14), moderately severe (15–19), and severe (20–27). Tested on a sample of 3000 primary care patients and 3000 obstetrics/gynaecology patients in the United States, the PHQ-9 has an internal reliability of 0.89 and a test-retest reliability of 0.84 across 48 h [28].

The GAD-7 is a 7-item self-report scale used to identify and measure the severity of generalised anxiety disorder. Scores range from 0 to 21, with a cut-off score of 8 or above distinguishing between clinical and non-clinical populations. Severity bands (and range of scores) are as follows: minimal/mild (0–9), moderate (10–14), and severe (15–21). Tested on a sample of 2739 primary care patients in the United States, the GAD-7 has an internal reliability of 0.92 and a test-retest reliability of 0.83 [29].

### Statistical analysis

Data were analysed using SPSS v 22 and MLwiN [30, 31]. Comparisons were made between CBT and counselling patients in terms of their intake severity levels of PHQ-9 and GAD-7 and their outcomes, pre-post change on PHQ-9 and recovery. The number of sessions attended by patients was also compared. Following this descriptive analysis, multilevel modelling (MLM) was utilised to model predictors of pre-post change.

### Criteria for determining recovery, improvement, no change, and deterioration

Analysis considered levels of clinical improvement and deterioration experienced by patients in the dataset using criteria of reliable and clinically significant change [21, 22]. Reliable and clinically significant improvement (RCSI) and associated change criteria are common indices of change in psychological therapy studies. Following the procedures set out by Jacobson and Truax [22], we considered patients had achieved RCSI if they (a) entered treatment in a dysfunctional state and left treatment in a normal state, and (b) having changed to a degree that was probably not due to measurement error, that is, by an amount equal to or greater than the reliable change index (RCI) described below.

We found an RCI for the PHQ-9 of 5.9 points, comparable to 6 points used by other researchers [22, 32]. Therefore, we also used an RCI of 6 or more point improvement to indicate reliable improvement and 6 or more point deterioration to indicate reliable deterioration. Clinical improvement can only be applied to patients above the clinical cut-off at intake – that is a score of 10 or more. Reliable change could therefore be applied to the full dataset, while reliable and clinically significant change could only be applied to patients clinical at intake.

### Multilevel modelling

Using iterative generalised least squares (IGLS) methods, a regression model was developed to identify significant patient variables, before including therapy type, the number of sessions attended and site effect. Continuous variables were added ‘grand mean centred’ to aid interpretation [24]. Therapy type, along with other patient variables, was deemed a statistically significant predictor of change if its coefficient was more than 1.96 times the standard error [31]. The significance of the improvement in the model fit, in the development from a single level model to a multilevel model, was considered by testing the difference in the −2*loglikelihood ratio produced by each model, against the chi squared distribution for the degrees of freedom of the additional parameters [31]. Using the estimates produced by the IGLS model as ‘priors’, Markov chain Monte Carlo (MCMC) procedures were applied. The size of the site effect was estimated as the percentage of the total variance that was at the site level and 95% Probability Interval (PrI) for this estimate was derived from the 2.5 percentile and 97.5 percentile values of the MCMC simulations chain [33].

## Results

First we present descriptive statistics regarding pre-therapy severity, the extent of change including rates of reliable and clinically significant change following psychological interventions and the number of sessions attended. We then focus on presenting an account of and results from the multilevel model development to consider potential predictors of outcome, including therapy type, and to estimate the size and significance of site effects.

### Descriptive statistics: Intake severity, pre- and post-therapy status and change

Table 1 presents the severity levels on the PHQ-9 for patients accessing CBT and counselling. Fewer mild and severe patients and more moderate and moderate-to-severe patients accessed counselling compared to CBT. With regard to the GAD-7, fewer severe patients (scoring 15–21) and more patients scoring 0–9 or 10–14 accessed counselling than CBT.

**Table 1:** Pre-therapy PHQ-9 scores and severity bands

PHQ-9 score and severity band	CBT % (N)	Counselling % (N)
0–9: Minimal/Mild	20.5 (5670)	19.8 (2265)
10–14: Moderate	21.7 (5994)	22.1 (2529)
15–19: Moderate-to-severe	27.0 (7467)	28.9 (3311)
20–27: Severe	30.9 (8550)	29.2 (3335)

For depression, the pre-therapy PHQ-9 mean (SD) for CBT was 15.4 (6.52) while for counselling it was also 15.4 (6.34). The mean (SD) pre-post change on PHQ-9 was 6.1 (6.96) for CBT and 5.9 (6.78) for counselling (*t* = 1.61 (18,361.8), *p* = 0.11). The post-therapy means (SD) were 9.3 (7.25) and 9.4 (6.98) respectively, yielding pre-post effect sizes (95% CI) of 0.94 (0.92, 0.95) for CBT and 0.95 (0.92, 0.98) for counselling.

The analysis was repeated for patients who scored above the clinical cut-off on PHQ-9 at intake. Here the pre-therapy PHQ-9 means (SD) for CBT and counselling were 17.9 (4.57) and 17.7 (4.56) respectively. The mean (SD) pre-post change was 7.3 (6.95) for CBT and 7.1 (6.71) for counselling (t(14,908.7) = 1.70, *p* = 0.09), giving post-therapy means (SD) of 10.6 (7.28) and 10.6 (7.02) and yielding pre-post effect sizes (95% CI) of 1.59 (1.58, 1.62) for CBT and 1.55 (1.52, 1.59) for counselling.

### Reliable and clinically significant improvement

For all patients attending services (i.e., clinical and non-clinical at intake), irrespective of the therapy received, 50.1% reached the criterion for reliable improvement on the PHQ-9, while 3.5% reliably deteriorated. The scores for the remaining 46.4% of patients did not reliably change. For CBT, 50.4% of patients made reliable improvement, 3.6% reliably deteriorated and 46.1% did not reliably change. The corresponding figures for counselling were 49.6%, 3.3% and 47.1%. Considering only patients scoring above the clinical cut-off at intake (*N* = 26,527), a total of 46.6% of patients receiving CBT and 44.3% of patients receiving counselling achieved reliable and clinically significant improvement on the PHQ-9.

### Sessions attended

**Fig. 2:**
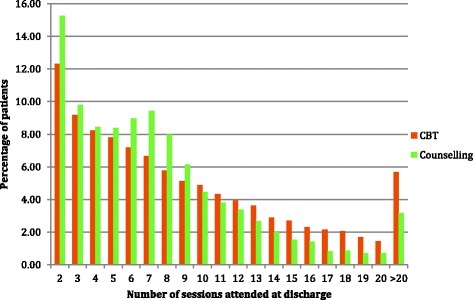
The number of sessions attended by CBT and counselling patients (*N* = 33,243)

Overall, the mean (SD) number of sessions attended was 8.5 (6.18), with a median (IQR) of 7 (4–12) sessions. For CBT, the mean (SD) was 8.9 (6.34) sessions, with a median (IQR) of 7 (4–12) sessions, while for counselling the figures were, 7.5 (5.54) sessions and 6 (3–10) sessions (Mann Whitney U, *p* < 0.001). Figure 2 presents the differences between the two therapies in terms of the number of treatment sessions patients had attended by the end of therapy and shows that patients receiving counselling generally had fewer sessions. For example, 74.4% of counselling patients had nine sessions or less compared with 62.3% of CBT patients.

### Multilevel model development

For the outcome ‘pre-post change in PHQ-9 scores’, a single level regression model identified patient intake severity on both PHQ-9 and GAD-7 and ‘Ethnicity’ as significant predictors of outcome. Adding therapy type, with CBT as the reference category, found that counselling reduced the amount of change on PHQ-9 by 0.195 (S.E. 0.080) of a point. However, this small but statistically significant amount was reduced and was no longer significant when ‘sessions attended’ was added to the model, although an interaction between number of sessions and therapy type was statistically significant. Intake PHQ-9 and sessions attended had non-linear relationships to pre-post change and both were included in the model as polynomial terms. Extending this model to a multilevel model significantly reduced the −2*loglikelihood ratio (*χ* ^2^(1) = 326.28, *p* < 0.001), indicating a significant improvement in model fit. Significant random slopes were found for intake PHQ-9 (*χ* ^2^(2) = 113.37, *p* < 0.001), and sessions attended, (*χ* ^2^(3) = 34.68, *p* < 0.001), indicating that the effect that intake PHQ-9 score and sessions attended had on patient outcomes varied between sites.

The full model derived using IGLS was developed using MCMC procedures. MCMC found that 20,000 iterations were sufficient for convergence of all estimates, and the testing of model assumptions using q-q plots and consideration of homoscedasticity indicated that normality can be assumed.

### The MCMC model

The final MCMC model (see Additional file 1) contained patient variables, therapy type and sessions attended, and the significant interactions between them. The model shows that three variables, PHQ-9 score at intake, the number of sessions attended, and the interaction between them, had positive coefficients (SE), of +0.485 (0.010), +0.287 (0.009) and +0.019 (0.001) respectively. With regard to PHQ-9 score, for each point above average on PHQ-9 at intake (15.4), change increased by just under half a point, while for each point below average at intake change reduced by the same amount. However, this is likely to be a statistical function due to higher intake scores having greater scope to change. Attending more sessions than average generally increased the amount of change by about a quarter of a point for each additional session, while patients who had above average intake scores and also attended above average number of sessions had a further small increase in change compared to patients who were above average on only one or neither of the variables.

Although, the modelling of the curvilinear relationship between intake score and change and also between sessions attended and change improved the model fit, the curves were slight and the coefficients for the squared terms small at −0.003 and −0.01 respectively. The effects though small indicate that for both variables, as values increase from the average to the maximum, the positive effect of increases is reduced and the reduction is greatest at the largest values. Conversely, the negative effect on change of below average intake scores and sessions attended is reduced as they decrease.

Higher than average GAD-7 scores at intake reduced change while lower scores increased change. The coefficient (SE) of −0.108 (0.009) indicates that for a maximum score on GAD-7 of 21, change on PHQ-9 would be reduced by about 0.7 of a point, while a GAD-7 score of zero would increase change by about 1.5 points. The coefficient (SE) for the interaction between PHQ-9 intake score and GAD-7 intake score, −0.01 (0.001), shows that higher than average intake scores on both PHQ-9 and GAD-7 reduced change on PHQ-9 compared to patients who were not above average on both, although the effect was small. Patient ethnicity was also a significant predictor, with ‘non-white British’ patients having about half a point less change, coefficient (SE) -0.536 (0.100), compared to ‘white British’ patients.

### Therapy type

The model found that therapy type was not a significant predictor of change on PHQ-9 with a coefficient (SE) for counselling of −0.073 (0.081) and a 95% PrI of −0.232 to +0.085. However, the small but statistically significant coefficient for the interaction between therapy type and sessions attended indicated that where patients receiving counselling had more than the average number of sessions, they tended to show less improvement than CBT patients with same numbers of sessions. For each session above average, counselling patients showed 0.066 of a point less change compared to CBT, but more change by the same amount per session where patients had less than the average number of sessions.

### Site effect

The model shows that although the amount of patient pre-post change on PHQ-9 for the average site was 6.729 points, it varied between sites, with a variance (SE) of 0.677 (0.123). The variance of residuals at the patient level was 35.716, therefore, the proportion of the total variance at the site level was 1.8%. MCMC indicated that this significant site effect had a 95% PrI of 1.3% - 2.6%.

The random slopes found for intake PHQ-9 score and sessions attended indicate that the effect that both variables have on outcome varied between sites. The variances (SEs) were small, 0.004 (0.001) and 0.003 (0.001) respectively, but the positive covariance (SE) between the intercept variance and the slope variance for PHQ-9 score, of 0.051 (0.010), describes a ‘fanning-out’ of the regression lines for sites. This indicates that as patient intake severity increased, there was greater variability between sites. Although the covariance (SE) between intercept variance and slope variance for sessions attended was also positive 0.010 (0.008), the ‘fanning-out’, that is the increase in variability between sites as number of sessions increased, was not significant.

**Fig. 3:**
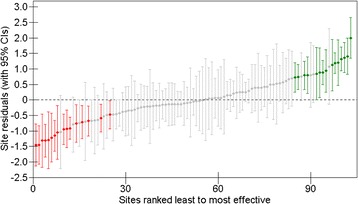
Caterpillar plot of the ranked residuals of 103 services with their 95% Confidence Intervals (CIs)

In Fig. 3, the variability between sites is described by ranking and plotting the 103 site residuals, derived from the model, with their 95% confidence intervals (CIs). As the model indicated, the average pre-post change for sites was 6.729 points but the site residuals show that the additional impact of each site varied from around −1.5 to +2.0 points. However, where the 95% CI for a site residual crosses zero, represented by the horizontal dashed line, the impact that the site has on outcomes is not significantly different to that of the average site. Accordingly, 72 (69.9%) sites, in grey, were not deemed to be significantly different to average, while 16 sites (15.5%) located to the left of the plot in red, were less effective than average and 15 (14.6%) sites on the right, in green, were more effective than average.

That the 95% confidence intervals of the ‘less effective than average’ and ‘more effective than average’ sites do not overlap would indicate that their impacts on change are significantly different. This is supported by comparisons between the three groups of sites in terms of their pre-post change on PHQ-9 in the full sample and recovery rates in the clinical sample. For the less effective sites, the mean pre-post change (SD) was 5.0 (0.68) points and the recovery rate (Range) was 42.7% (32.0% - 48.0%), while for more effective sites, the mean pre-post change (SD) was 7.0 (1.06) points and the recovery rate (Range) was 58.7% (51.4% - 66.3%). The mean pre-post change (SD) for average sites was 6.0 (1.09) while the recovery rate was 51.0% with a wide range (19.4% - 72.2%) that included the sites with the lowest and highest recovery rates overall. These two sites can be seen in Fig. 3 as the lowest and highest ranked average (grey) sites, classified as average by the multilevel model due to case-mix adjustments and the wide 95% confidence intervals for their estimated residuals.

### Therapy type, recovery and sessions

**Fig. 4:**
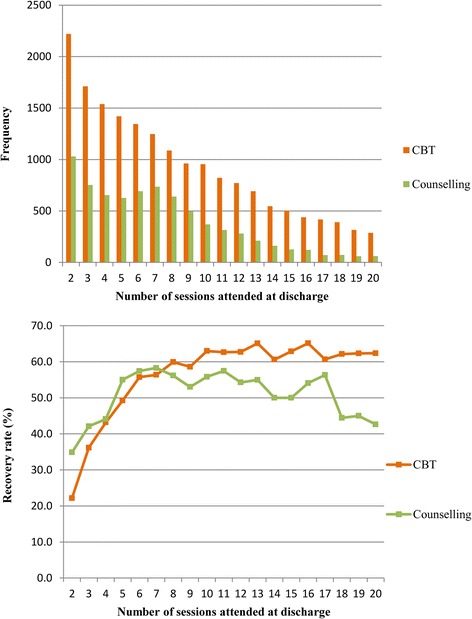
Number of sessions attended (2–20) and recovery rates (Clinical sample: *N* = 26,527)

As the model indicated, where patients had more than the mean number of sessions (8.5), CBT was slightly more effective. In order to consider further the relationships between therapy type, sessions attended and outcomes, Fig. 4, compares recovery rates for CBT and counselling for patients who received 2–20 sessions, and also shows the numbers of patients included in the calculation of rates. In total, 94.8% of patients who scored in the clinical range at intake had between 2 and 20 sessions.

Figure 4 shows that for 2 to 7 sessions (representing 54% of patients), counselling had higher recovery rates while for 8 or more sessions CBT had higher rates and the difference in rates appears to widen as the number of sessions increases. However, as the number of sessions increases, the recovery rates were based on fewer patients, particularly for counselling. Consideration of 95% CIs around these recovery rates indicated that only where patients had either 18 or 20 sessions was CBT significantly more effective, with recovery rates (95% CIs) of 62.2% (57.1, 66.9) and 62.4% (56.5, 68.0) respectively, compared with 44.4% (32.7, 56.6) and 42.6% (30.0, 55.9) for counselling. The confidence intervals of the recovery rates overlapped for all other sessions, apart from where patients attended only two sessions. Where patients attended two sessions, counselling was significantly more effective, with a recovery rate (95% CI) of 34.9% (31.9, 37.9) compared with 22.2% (20.5, 24.0) for CBT.

## Discussion

### Comparative effectiveness of CBT and counselling

The current study utilised a large dataset comprising in excess of 30,000 patients seen at over 100 sites and collected as part of a national audit of psychological therapies and found the outcomes of CBT and generic counselling to be comparable and that the model of therapy did not predict outcome. By contrast, there was significant variability between sites, with approximately 15% of sites yielding reliably more effective patient outcomes and a similar percentage yielding reliably less effective outcomes. The mean recovery rates for these two groupings were 59% and 43% respectively and the most effective site had a recovery rate more than twice that of the least effective site. Importantly, in the current study we employed multilevel modelling to capture the nested nature of routinely collected service level data and also controlled for patient variables and sessions attended. Hence, if the less effective sites were treating more severe patients, this was accounted for in the analysis.

Accordingly, our findings strongly suggest that, despite the very different recommendations for CBT and counselling in the NICE Guidelines for Depression in Adults [3], it would appear that the two therapies have a very similar impact in routine practice for the treatment of depression. Indeed, as observed in the current data, CBT only had significantly better outcomes than counselling for patients attending 18 and 20 sessions, which only accounted for 3.2% of the patients who attended up to the 20 sessions. By contrast, for 12.9% of patients who attended two sessions, counselling was significantly more effective. However, for the vast majority of patients (i.e., 83.9%) there were no significant differences in outcomes between the two therapies and hence no rationale for suggesting CBT to be superior to generic counselling in the treatment of depression.

Our results contrast with findings from previous research that structured and focused interventions such as CBT are more efficacious than unstructured non-specific interventions, of which counselling is often categorised [6]. Furthermore, it has been reported that when psychological therapy is efficacious, post-treatment effect sizes are higher for high-severity patients than for low-severity patients [34]. It could be inferred that non-specific therapies (i.e., counselling) may be less efficacious for high-severity patients than a specific therapy (i.e., CBT). Our findings do not support this hypothesis. In line with the findings reported here, when counselling as delivered within the UK primary healthcare setting of IAPT services has been directly compared to more structured psychological interventions such as CBT, the two interventions have been consistently reported to be comparable [8, 10]. To further test this proposition, a large scale randomised controlled trial comparing the efficacy and cost-effectiveness of Counselling for Depression with CBT as delivered within an IAPT service is currently underway [35]. Using a non-inferiority analysis this trial will enable a comparison of a specific manualised form of counselling to be compared to CBT in a sample of patients presenting with moderate or severe depression.

The findings reported here extend and refine previous evaluations from IAPT data that suggested counselling and CBT are equally effective in the treatment of depression [15, 19, 20]. Similar findings of broadly equivalent outcomes for depression have been reported from the analyses of other large UK routine practice datasets that pre-dated the IAPT initiative [6, 11]. Taking a perspective that considers the weight of evidence rather than just RCT methodology, there seems little evidence for NICE guidance to continue to suggest or label counselling as a second line intervention for the treatment of adult depression.

### Variability between sites

Although it is clear the type of therapy provided to patients with depression has little differential impact on outcomes, there are other factors that may have a greater role in whether or not a patient responds well to psychological therapy. The results presented here describe the variability between sites in terms of outcomes for patients, with some sites yielding significantly better outcomes than others after controlling for patient variables, therapy type and sessions attended. This finding is consistent with other accounts showing large variability in outcomes across IAPT sites [16]. Our results suggest that if the 16 less effective sites had outcomes similar to average sites, then a further 364 patients would have recovered across those sites in in a 4-month time period.

Previous research has also identified variability in another key service variable – therapists – as an important factor for patient outcomes [36, 37]. A recent study of a single IAPT service, included but not identifiable in the NAPT audit, yielded a therapist effect of 5.8% [38]. While the data available in the present study did not allow exploration of therapist effects, the growing evidence for variability of sites and therapists suggests a need for a move away from debates about the superiority of one brand of therapy over another and towards a more comprehensive understanding of the factors that contribute to some services – and therapists – yielding more effective patient outcomes than others and, more importantly, understanding why some sites are yielding less effective patient outcomes. Socio-economic status and intake severity have been reported to impact on outcomes [39–41]. It is possible that some services have a higher proportion of complex cases or of unmeasured patient factors in their populations that would account for poorer overall outcomes.

### Efficiency

Although overall there was no difference in outcomes between the two therapies, and a significant difference at only 2, 18 and 20 sessions, our findings suggest that CBT tends to be more effective at 8 or more sessions while counselling tends to be more effective at less than 8 sessions. Given that the majority of patients in IAPT are being treated in fewer than ten sessions, this finding is of some significance and warrants further investigation as it could be argued that counselling is more efficient than CBT in treating depression. Such a finding could have positive cost implications for the NHS.

### Caveats

There are a number of caveats to this study. First, although the study did not sample all IAPT services, an approximate 50% data capture of all services suggests a broadly representative sampling frame. In addition, the ratio of patient numbers for CBT and counselling in the sample (2.2:1) is similar to that reported in the 2013/14 HSCIC report (2.4:1) [15]. Second, a standard criticism of data captured by these methods being used to make treatment comparisons is that there is no randomisation of patients and no independent fidelity checks that the therapies as labelled would meet respective quality checks. While the feasibility of such independent checks is doubtful in routine practice, there are standards of supervision for the delivery of therapies in IAPT that are assumed to have been adhered to by all services. In addition, there is no reason to believe there is any effect that would systematically favour or weaken one of the interventions over the other. However, a randomised non-inferiority trial would be required to provide a more definitive answer to the question posed here.

Third, the data comprises only pre- and post-therapy data and so the number of sessions required to meet clinical change was calculated based on the point at discharge. Not available in the dataset was the weekly PHQ-9 score recorded at each session. Analyses of this data might have yielded evidence showing that patients met the required threshold in fewer sessions than reported in this study. However, given the sessional data is mandated by the IAPT services and was available to the clinicians, there is no reason to suppose that the point of discharge would have been any different than that reported in this study. Finally, while the NAPT data captured service-level data, it did not collect therapist level data. Nesting patients within therapists, who are nested within sites in a three level model would have allowed the estimation of both therapist effects and site effects and the contribution both make to patient outcomes. There is a need for further studies of large datasets that contain and identify the three levels.

## Conclusions

It is apparent from the findings presented here that counselling is not inferior to CBT and there would seem little, if any, rationale for committing public money to fund superiority trials of CBT in the field of depression. Instead, attention needs to be focused on factors other than therapy type that may influence outcomes, namely the factors associated with the variability between services including therapists. Such attention should adopt multilevel modelling as the analytic approach in order to capture the naturally nested nature of the delivery of psychological therapies [42]. However, future research aims to explore the efficacy of non-CBT psychological therapies using a randomised controlled trial design, adopting CBT as the comparator condition in order to build the evidence base for alternate bona fide therapies and enable greater provision and choice for patients. There is also a need to undertake more thorough analyses of existing datasets, particularly the routine data collected by IAPT to take account of the issues raised in this paper. Finally, in the context of the very small differences between psychological interventions, what is striking from the results in the current audit is that the scores of approximately half of all patients, regardless of the intervention received, either did not achieve reliable improvement or reliably deteriorated. It is a salutatory reminder of the extent of work remaining to be done regarding the implementation and delivery of effective psychological therapies and where efforts might better be focused rather than on pursing research into insignificant differences between the effectiveness of differing models of psychological interventions.

## Additional file

Additional file 1: Multilevel model, using MCMC, for pre-post change on PHQ-9. (DOCX 37 kb)

## References

[1] Layard R, Clark D, Bell S, Knapp M, Meacher B, Priebe S (2006). The depression report: a new deal for depression and anxiety disorders.

[2] Department for Health. IAPT three-year report. The first million patients. November 2012. http://www.mhinnovation.net/sites/default/files/downloads/innovation/reports/Three-year-report.pdf. Accessed 9 Apr 2017.

[3] NICE. Depression in adults: The treatment and management of depression in adults. NICE clinical guideline 90; 2009. https://www.nice.org.uk/guidance/CG90. Accessed 9 Apr 2017.

[4] Bower P, Rowland N, Hardy R. The clinical effectiveness of counselling in primary care: A systematic review and meta-analysis. Psychol Med. 2002;33:203–15. 10.1037/0022-0167.33.1.23 10.1017/s0033291702006979 12622300

[5] Propst LR, Ostrom R, Watkins P, Dean T, Mashburn D (2002). Comparative efficacy of religious and nonreligious cognitive–behavioral therapy for the treatment of clinical depression in religious individuals. J Consult Clin Psychol.

[6] Cuijpers P, Driessen E, Hollon SD, van Oppen P, Barth J, Andersson G. The efficacy of non-directive supportive therapy for adult depression: a meta-analysis. Clin Psychol Rev. 2012;32:280–91. 10.1016/j.cpr.2012.01.003 22466509

[7] Cuijpers P, Straten A, Andersson G, Oppen PC (2008). Psychotherapy for depression in adults: a meta-analysis of comparative outcome studies. J Consult Clin Psychol.

[8] Cape J, Whittington C, Buszewicz M, Wallace P, Underwood L. Brief psychological therapies for anxiety and depression in primary care: meta-analysis and meta-regression. BMC Med. 2010;8:38. 10.1186/1741-7015-8-38 PMC2908553 20579335

[9] Cuijpers P, Sijbrandij M, Koole SL, Andersson G, Beekman ATF, Reynolds CF (2013). The efficacy of psychotherapy and pharmacotherapy in treating depressive and anxiety disorders: a meta-analysis of direct comparisons. World Psychiatry.

[10] Ward E, King M, Lloyd M, Bower P, Sibbald B, Farrelly S, et al. Randomised controlled trial of non-directive counselling, cognitive-behaviour therapy, and usual general practitioner care for patients with depression. I: Clinical Effectiveness. Brit Med J. 2000;321:1383–8. 10.1136/bmj.321.7273.1383 PMC27542 11099284

[11] King M, Marston L, Bower P. Comparison of non-directive counselling and cognitive behaviour therapy for patients presenting in general practice with an ICD-10 depressive episode: a randomized control trial. Psychol Med. 2014;44:1835–44. 10.1017/S0033291713002377 24103190

[12] MacPherson H, Richmond S, Bland, Brealey S, Gabe R, Hopton A, et al. Acupuncture and counselling for depression in primary Care: a randomised controlled trial. PLoS Med. 2013;10:e1001518. 10.1371/journal.pmed.1001518 PMC3782410 24086114

[13] Sanders P, Hill A. Counselling for depression. A person-centred and experiential approach to practice. Sage; 2014.

[14] Pearce P, Sewell R, Hill A, Coles H, Pybis J, Hunt J, et al. Counselling for depression: the perceptions of trainees. Healthc Couns Psychother J. 2013:8–13.

[15] Health and Social Care Information Centre (HSCIC). Improving Access to Psychological Therapies report. 2015. http://www.hscic.gov.uk/catalogue/PUB17755. Accessed 9 Apr 2017.

[16] Digital NHS (2016). Psychological therapies: annual report on the use of IAPT services.

[17] Report of the second round of the National Audit of Psychological Therapies. Royal College of Psychiatrists. 2013. http://www.rcpsych.ac.uk/pdf/NAPT%20second%20round%20National%20report%20%20website%2028-11-13v2.pdf. Accessed 9 Apr 2017.

[18] Perfect D, Jackson C, Pybis J, Hill A. Choice of therapies in IAPT: An overview of the availability and client profile of step 3 therapies, BACP. 2016. http://www.bacp.co.uk//docs/pdf/15641_napt_v6.pdf.Accessed 9 Apr 2017.

[19] Glover G, Webb M, Evison F (2010). Improving access to psychological therapies: a review of the progress made by sites in the first roll-out year.

[20] Gyani A, Shafran R, Layard R, Clark DM. Enhancing recovery rates in IAPT services: Lessons from analysis of the year one data. 2011. https://www.ncbi.nlm.nih.gov/pmc/articles/PMC3776229/. Accessed 9 Apr 2017.

[21] Evans C, Margison F, Barkham M (1998). The contribution of reliable and clinically significant change methods to evidence-based mental health. Evid Based Ment Health.

[22] Jacobson NS, Truax P. Clinical significance: A statistical approach to defining meaningful change in psychotherapy research. J Consult Clin Psychol. 1991; doi.org/10.1037/0022-006X.59.1.12 10.1037//0022-006x.59.1.12 2002127

[23] National Audit of Psychological Therapies for anxiety and depression. Royal College of Psychiatrists. 2011. http://www.rcpsych.ac.uk/pdf/NAPT%202011%20Report%20.pdf. Accessed 9 Apr 2017.

[24] Snijders TAB, Bosker RJ (2012). Multilevel analysis: an introduction to basic and advanced multilevel modelling. 2nd ed.

[25] Rasbash J, Charlton C, Browne WJ, Healy M, Cameron B. MLwiN Version 2.1. Centre for Multilevel Modelling, University of Bristol; 2009.

[26] Department for Health. Which talking therapy for depression? A guide to understanding the different psychological therapies you may be offered to treat your depression n.d.

[27] British Association for Counselling and Psychotherapy http://www.bacp.co.uk/. Accessed 9 Apr 2017.

[28] Kroenke K, Spitzer RL, Williams JB (2001). The PHQ-9: validity of a brief depression severity measure. J Gen Intern Med.

[29] Spitzer RL, Kroenke K, Williams JB, Löwe B. A brief measure for assessing generalized anxiety disorder: the GAD-7. Arch Intern Med. 2006;166:1092–7. 10.1001/archinte.166.10.1092 16717171

[30] IBM Corp. Released 2013. IBM SPSS statistics for windows, version 22.0. Armonk, NY: IBM Corp.

[31] Rasbash J, Steele F, Browne WJ, Goldstein H. A user’s guide to MLwiN, v2.26. Centre for Multilevel Modelling, University of Bristol; 2012.

[32] Stiles WB, Barkham M, Mellor-Clark J, Connell J. Effectiveness of cognitive-behavioural, person-centred, and psychodynamic therapies in UK primary-care routine practice: replication in a larger sample. Psychol Med.2008;38:677–88. 10.1017/S0033291707001511 17825124

[33] Browne WJ MCMC estimation in MLwiN Version 2.13. Centre for Multilevel Modelling, University of Bristol; 2009.

[34] Driesson E, Hollon SD, Cuijpers P, Dekker JJM. Does pretreatment severity moderate the efficacy of psychological treatment of adult outpatient depression? A meta-analysis. J Consult Clin Psychol. 2010;78:668–80. 10.1037/a0020570 20873902

[35] Saxon D, Ashley K, Bishop-Edwards L, Connell J, Harrison P, Ohlsen S, et al. A pragmatic randomised controlled trial assessing the non-inferiority of counselling for depression versus cognitive-behaviour therapy for patients in primary care meeting a diagnosis of moderate or severe depression (PRaCTICED): study protocol for a randomised controlled trial. Trials.2017;18:93. 10.1186/s13063-017-1834-6 PMC5333411 28249592

[36] Kim DM, Wampold B, Bolt DM. Therapist effects in psychotherapy: a random-effects modeling of the National Institute of Mental Health Treatment of Depression Collaborative Research Program data. Psychother Res. 2006;16:161–72.

[37] Okiishi JC, Lambert MJ, Eggett D, Nielson L, Dayton DD, Vermeersch DA (2006). An analysis of therapist treatment effects: Towards providing feedback to individual therapists on their clients’ psychotherapy outcome. J Consult Clin Psychol.

[38] Saxon D, Firth N, Barkham M. The relationship between therapist effects and therapy delivery factors: therapy modality, dosage, and non-completion. Adm Policy Ment Health. First Online: 16 July 2016. doi:10.1007/s10488-016-0750-5. 10.1007/s10488-016-0750-5 PMC5550525 27424106

[39] Garfield SL. Research on client variables in psychotherapy. In AE Bergin & SL Garfield (Eds.), Handbook of psychotherapy and behavior change. 4th ed. New York: Wiley; 1994. p. 190–228.

[40] Ostler K, Thompson C, Kinmonth AL, Peveler RC, Stevens L, Stevens A (2001). Influence of socio-economic deprivation on the prevalence and outcome of depression in primary care: the Hampshire depression project. Br J Psychiatry.

[41] Luborsky LML, Diguer AT, Woody L, Seligman G, DA. (1997). The psychotherapist matters: comparison of outcomes across twenty-two therapists and seven patient samples. Clin Psychol Sci Pract.

[42] Saxon D, Barkham M (2012). Patterns of therapist variability: therapist effects and the contribution of patient severity and risk. J Consult Clin Psychol.

