# Supplementary figures and images for: The comparative effectiveness and efficiency of cognitive behaviour therapy and generic counselling in the treatment of depression: evidence from the 2nd UK National Audit of psychological therapies
Source: BMC Psychiatry. 2017 Jun 9;17:215. doi: 10.1186/s12888-017-1370-7 (PMC5466727; doi:10.1186/s12888-017-1370-7)

Additional file 1. Multilevel model, using MCMC, for pre-post change on PHQ-9


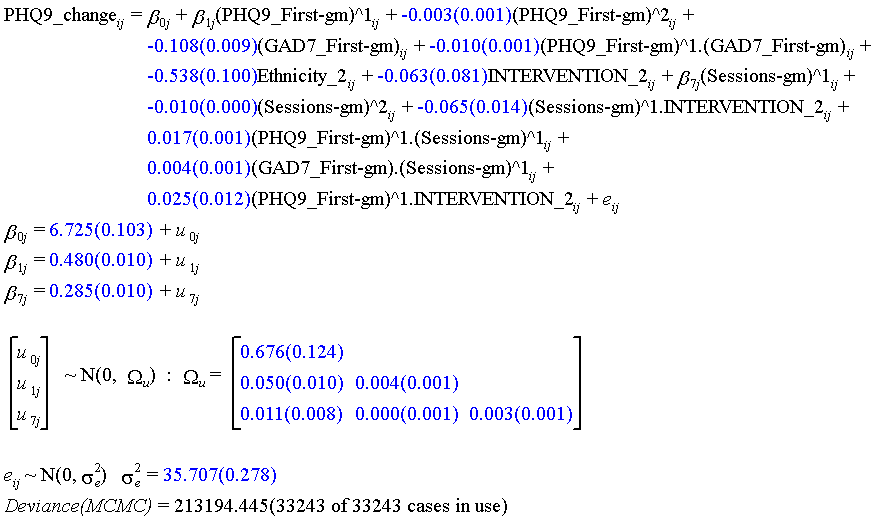

Supplement: Additional file 1: — Multilevel model, using MCMC, for pre-post change on PHQ-9. (DOCX 37 kb) [file 12888_2017_1370_MOESM1_ESM.docx]
